# Protective Role of Kynurenine 3-Monooxygenase in Allograft Rejection and Tubular Injury in Kidney Transplantation

**DOI:** 10.3389/fimmu.2021.671025

**Published:** 2021-07-07

**Authors:** Randi Lassiter, Todd D. Merchen, Xuexiu Fang, Youli Wang

**Affiliations:** ^1^ Department of Surgery, Medical College of Georgia at Augusta University, Augusta, GA, United States; ^2^ Division of Nephrology, Department of Medicine, Medical College of Georgia at Augusta University, Augusta, GA, United States

**Keywords:** kidney transplantation, kynurenine 3-monooxygenase, allograft rejection, kynurenine metabolism, immunosuppresant

## Abstract

Renal tubular epithelial cells (TECs) are the primary targets of ischemia–reperfusion injury (IRI) and rejection by the recipient’s immune response in kidney transplantation (KTx). However, the molecular mechanism of rejection and IRI remains to be identified. Our previous study demonstrated that kynurenine 3-monooxygenase (KMO) and kynureninase were reduced in ischemia–reperfusion procedure and further decreased in rejection allografts among mismatched pig KTx. Herein, we reveal that TEC injury in acutely rejection allografts is associated with alterations of Bcl2 family proteins, reduction of tight junction protein 1 (TJP1), and TEC-specific KMO. Three cytokines, IFN**γ**, TNF*α*, and IL1β, reported in our previous investigation were identified as triggers of TEC injury by altering the expression of Bcl2, BID, and TJP1. Allograft rejection and TEC injury were always associated with a dramatic reduction of KMO. 3HK and 3HAA, as direct and downstream products of KMO, effectively protected TEC from injury *via* increasing expression of Bcl-xL and TJP1. Both 3HK and 3HAA further prevented allograft rejection by inhibiting T cell proliferation and up-regulating aryl hydrocarbon receptor expression. Pig KTx with the administration of DNA nanoparticles (DNP) that induce expression of indoleamine 2,3-dioxygenase (IDO) and KMO to increase 3HK/3HAA showed an improvement of allograft rejection as well as murine skin transplant in IDO knockout mice with the injection of 3HK indicated a dramatic reduction of allograft rejection. Taken together, our data provide strong evidence that reduction of KMO in the graft is a key mediator of allograft rejection and loss. KMO can effectively improve allograft outcome by attenuating allograft rejection and maintaining graft barrier function.

## Highlights

Immunosuppressants reduce allograft rejection to prolong allograft survival in kidney transplantation. However, the long-term use of these medications often results in complications that can lead to diseases or even allograft loss. Thus, endogenous immunosuppressive metabolites may represent more ideal medical therapies. Indoleamine 2,3-dioxygenase (IDO) has been reported to prevent allogeneic fetal rejection. To date, neither the molecular mechanism of tolerance induced by IDO nor its downstream enzymes have been clearly defined. This paper demonstrates that kynurenine 3-monooxygenase (KMO), a downstream enzyme of IDO, is the main player in tolerance induced by the kynurenine metabolites, as its direct and downstream products, 3HK and 3HAA, can prevent T cell-induced allograft rejection and protect the tubular epithelial cell from injury caused by the cytokine storm formed in ischemia–reperfusion and early allograft rejection procedure.

## Introduction

Kidney transplantation (KTx) is the best treatment for patients with renal failure. Current KTx requires overcoming two major obstacles, including the shortage of available organs for transplant and significant side effects of antirejection medications. Scientists are developing novel organ resources for clinical transplantation. Among all, swine are the most ideal organ donor for clinical transplants due to: (i) the similar size of kidneys and ease of transplantation; (ii) similar renal metabolic function for unrestricted food intake and metabolism; (iii) a variety of swine leukocyte antigens that offer biological diversity and antigen-driven rejection mechanisms similar to humans. Additionally, researchers are also using blastocyst complementation combined with gene editing to increase organs favorable for human transplant (1–3). Although pigs can provide enough organs for transplantation, there is still some distance from the laboratory bench to clinical application. The major barrier is the transmission of infectious microorganisms from pigs to humans (4–9). Although many studies have reported that there are no transmissions of virus in xenotransplantation (10–13), inactivation of endogenous porcine retroviruses using CRISPR-Cas9 technology makes xenotransplantation safer and potentially possible in the future (14, 15).

Rejection of the recipient’s immune system to imported allografts forms the main barrier for successful clinical transplantation and future xenotransplantation (16, 17). Currently, the combination of immunosuppressants to deactivate the recipient’s immune response is used to prevent rejection. These immunosuppressants can temporarily inhibit acute rejection to prolong an allograft’s survival but have less impact on long-term allograft outcomes. The toxicity of immunosuppressants causes complications leading to new diseases that can ultimately lead to graft loss. Endogenous metabolites with antirejection properties may make ideal drugs owing to less toxicity and higher specificity.

Indoleamine 2, 3-dioxygenase (IDO) is the first enzyme in the metabolic pathway from which tryptophan is metabolized to kynurenines. IDO has been shown to prevent allogeneic fetal rejection (18). This antirejection specifically relies on tryptophan depletion and the production of kynurenines (19, 20). IDO downstream enzymes, kynurenine 3-monooxygenase (KMO) and kynureninase produce kynurenine derivatives 3HK (hydroxyl-3 kynurenine) and 3HAA (hydroxyl-3 anthranilic acid), which have been shown to effectively inhibit T cell proliferation (21), thus improving tolerance (22–26). Although induced IDO can increase tolerance in some rodent transplantation models (18, 26–35), our recent study and other scientists’ observations have indicated that IDO, itself, only predicts allograft rejection (36–40). Using our own porcine kidney transplant model, we showed that rejection allografts was associated with down-regulation of KMO and kynureninase and up-regulation of IDO (40).

Tubular epithelial cell (TEC) injury, not glomerular injury, is the primary target of the recipient’s immune system during acute rejection (41, 42). Ischemia–reperfusion, an unavoidable part of the KTx procedure, can also cause ischemia–reperfusion injury (IRI) to TEC. This has been identified through three key signaling pathways (43). Furthermore, a 1-h extension of cold ischemia time increases the risk of rejection by 4% post-transplant (44, 45). Our previous study revealed that alterations of cytokine expression in early allograft rejection were associated with an increase in IDO and a decrease in KMO and other kynurenine metabolic enzymes (40). In the present study, we investigate the influence of cytokine exposure on the gene expression of kynurenine metabolites and cell injury proteins in human primary TEC and allograft rejection. A crucial role of KMO and kynureninase in preventing TEC injury and attenuating allograft rejection will be demonstrated.

## Materials and Methods

### Animal Study

Studies were performed on 30–40 kg outbred female Yorkshire piglets (Palmetto Research Swine, Reevelville, South Carolina) as we have described previously (40). Briefly, one pair of pigs was operated simultaneously such that the left kidneys were exchanged (allotransplants) or retransplanted (autotransplants). All transplants were *ex vivo* perfused at 4°C and orthotopically transplanted, followed by right nephrectomy and closure (40). No immunosuppressants were used. All auto- and allotransplanted kidneys and other physiological samples were collected 72 h post-transplantation from the live animals before euthanasia. These studies were approved by Augusta University Institutional Animal Care and Use Committee. Tissues from collected organs were processed for future examination of histology, enzymatic assay, protein expression, and mRNA alterations.

### Murine Skin Transplantation

Murine skin transplantation was performed with a modification of the previous protocol (46). Briefly, mouse-ear skin was utilized as a graft onto the back of a recipient mouse. Balbc and C57/B6 mice between 10 and 13 weeks old were used in syngeneic and allogeneic transplants. Rather than suturing, the graft was held in place with a four layer flexible bandage which remained in place in a standard length of time until dressing removal (Fang X, unpublished).

### Human Primary Cell Culture

Human cortical renal TEC (HREC, #CC-2554, Lonza) was cultured with a specific medium (#CC-3190, Lonza). TEC expansion, passage, and stocking were performed by following vendor protocols (#CC-5034, Lonza). Cells were seeded in a 1:3 split in six-well plates and grown to 85% confluency with media changes every other day. Then cells were replaced with fresh media and pretreated in the presence or absence of 5–100 μM 3HK (Sigma) or 10–40 μM 3HAA (Sigma) for 16 h. Then cells were challenged with or without cytokine cocktail (15 nM of IFN*γ* + 6 nM of TNF*α* + 3 nM of IL1β) for 24 h prior to harvesting. Trizol was utilized for RNA isolation and assays. RIPA buffer was used to lyse cells for Western blot.

### Human Peripheral Blood Pan-T Cell Proliferation

Human peripheral blood Pan-T cells (hPBTs, Stemcell Technologies, Canada) were grown in ImmunoCult™-XF T cell expansion medium plus ImmunoCult™-human CD3/CD28/CD2 (25 µl/ml, StemCell Technologies, Canada) and IL-2 (10 ng/ml, StemCell Technologies, Canada) for 4 days. Following this period of expansion/activation, the activated hPBTs were labeled with CellTrace™ Cell Proliferation Kits (#C34557, Invitrogen). Labeled or unlabeled activated hPBTs were grown in proliferation assay medium [PAM, ImmunoCult™-XF T cell expansion medium plus ImmunoCult™-Human CD3/CD28/CD2 (25 µl/ml)] and IL2 (2 ng/ml) in the presence of 3HK. The cells were collected and washed with PBS, and then the cells were fixed with 1% paraformaldehyde in PBS for 20 min at room temperature. The proliferation of T cells was determined with flow cytometry.

### Histology and Immunostaining

Freshly dissected kidneys were fixed overnight with 4% paraformaldehyde in PBS (pH 7.2) at 4°C, and kidneys were cut into 4-μm sections. The immunostaining procedure was performed as described previously (40). Immunochemistry was completed using a KMO antibody (Novus Biologicals, Cat.NBP1-86335) and Cyclophilin A (Abcam. Cat. Ab154388) with 1:400 dilutions in 5% donkey serum in standard TBS buffer. The staining was viewed with color developed from horseradish peroxidase complex (Vector Laboratories) and counterstained with hematoxylin.

### Quantitative RT-PCR

Quantitative RT-PCR was carried out as previously described (40, 47). Total RNA was isolated from renal tissue and cultured cells with TRIZOL. The RNA was converted to cDNA using SuperScript III First-Strand Synthesis System (Invitrogen). PCR primers producing 100–130 bp amplicon were designed using Primer 3 Input software, the primers for candidate genes were shown in **Table 1**, and quantitative PCR was performed with SsoAdvanced Universal SYBR Green Kit (Bio-Rad Lab) on a CFX Connect real-time PCR detection system (Bio-Rad Lab). *β*-actin mRNA was used for normalization.

**Table 1:** Primers used for qPCR.

Gene Name	Forward Primer	Reverse Primer
β-actin	gtcaccaactgggacgacat	tcttctcacggttggctttg
IDO1	gcacatctggttctggggta	gaggcagtccaagcttctca
KMO-1	atcgcctgtgacctcatctt	aacttcatgtagccgtgagg
KMO-2	aatttgcacgtggaagaagc	tggggataccttgggataca
KMO-3	aagataccatgaggccatgc	ccggtaaggtaggtggt
DDC	ctggagacagtgatgatggact	aaggtagcttcactggcacttc
HAAO	cctatgagacccaggtaatcgt	ctctccttccattgtcaccact
ACMSD	gtccaagagaactgctggaatc	gaagctggcacaggtctaaagt
Bcl-xL	ggtattggtgagtcggatcg	tctcagctgctgcattgttt
TJP1	tcgcattgtagagtcggatg	ccacgacacggaatacctct
TJP2	gagacaacccccactttgaa	accacccgatcattttcttg
N-cadherin	cctcgtcagagaccacctgt	ggcatatgtcgccagagaat
IL6	ttcacctctccggacaaaac	tctgccagtacctccttgct
IL17	gacggccctcagattactcc	ttccttcccttcagcattga
IL18	ctgctgaaccggaagacaat	aggttcaagcttgccaaagt

ACMSD, Aminocarboxymuconate-semialdehyde decarboxylase; DDC, dopa decarboxylase; N-cad, N-cadherin; HAAO, 3-hydroxyanthranilate 3,4-dIoxygenase; TJP1, tight junction protein; TJP2, tight junction protein 2; Bcl-xL, B-cell lymphoma-extra large.

### TUNEL Staining

TUNEL staining was performed with the ApopTag Plus Peroxidase *In Situ* Apoptosis Detection Kit (Millipore S7101) according to the manufacturer’s protocol. TUNEL-positive cells were randomly counted from five different 20× microscopic vision fields for each pig. Total TUNEL-positive cells were quantified from the tissues of four to eight pigs per group.

### LDH Assay

3HK toxicity to TEC, tubular endothelial cells (TEndo), and hPBT were assessed with lactate dehydrogenase (LDH) assays using Pierce LDH Cytotoxicity Assay Kit and the chemical compound-mediated cytotoxicity assay protocol (Thermo Scientific). Briefly, TEC, TEndo, and hPBT were grown in their growth media to 85% confluency, while hPBT was grown in the mixture of TEC media and hPBT media in a 2:1 ratio to reduce hPBT medium background in the LDH assay. TEC, TEndo, and hPBT were then treated with different doses of 3HK. LDH assay was performed by following the manufacturer’s instructions.

### Western Blot

Renal tissue and cells were lysed in RIPA buffer containing the cocktail of proteinase inhibitors (Roche) and phosphatase inhibitors. Protein concentrations were determined using a bicinchoninic acid protein assay reagent kit (Pierce). Proteins were separated by SDS-PAGE, and Western blotting was carried out as previously described (40). The following antibodies were used as probes for Western blotting: *β*-actin (Sigma, A5316); KMO (NBP1-86335), kynureninase (AF4887-SP), alpha-smooth muscle actin (NB300-978SS), snail (NBP2-27184SS), E-cadherin (NBP2-19051SS), tight junction protein 1 (NBP1-85047) were from Novus Biologicals; Bax (sc-7480), Bcl-xL (sc-8392), BID (sc-373939), caspase 3 (sc-7272), caspase 8 (sc-56070), VDR (sc-1008), AhR (sc-133088), pNF*κ*B65-S536 (sc-136548), NF*κ*B65 (sc-8008), ERK-Th202/Ty204 (sc-136521), MHCI (sc-55582), ERK (sc-514302) were from Santa Cruz Biotechnology. Akt-S473 (9271S), Akt (9272S), pPTEN-S380 (9551), and PTEN (9552), MHCII (LGII-612.14), and Bcl2 (4223T) were from Cell Signaling; IDO1 (M256) was obtained from CalBioreagents. Secondary antibodies were horseradish peroxidase-conjugated anti-rabbit or anti-mouse IgG (Pierce Biotechnology), and signals were detected using SuperSignal West Pico or Dura Extended Duration Substrate (Pierce). *β-*actin was used for loading normalization. The relative amount of each protein was quantified using NIH ImageJ software.

### DNA Nanoparticles

DNA nanoparticles (DNP) were prepared as previously described (48). Briefly, 21 µg of pCpG free–Lacz DNA (InvivoGen, CA) was mixed with 200 µl of 10% of D-(+)-glucose solution at room temperature. Then 7 µl of 150 mM polyethylenimine (PEI, Polysciences Inc, PA) was mixed with another 200 µl of 10% of D-(+)-glucose solution in room temperature. The DNA–glucose solution was then added to PEI–glucose solution under gentle vortex. The mixture of PEI–DNA was kept at room temperature for 10–15 min to form DNA complex. Twenty-one micrograms of DNA in 0.4 ml 10% glucose solution was intravenously injected into 20 g mice or *ex vivo* perfused into 20 g of pig kidney. For the kidneys larger than 20 g, the ratio of DNA micrograms per PEI in microliters was maintained at 3:1. Thus the amount of DNA, PEI, and 10% glucose solution increased proportionately in accordance with the increase in kidney weight.

### Statistical Analyses

Data were presented as means ± SEM. Statistical comparisons were carried out using unpaired t-test or one-way ANOVA as appropriate, with p <0.05 considered as statistically significant

## Results

### Pathology and Gene Expression of Grafts in Pig Kidney Transplantation

**Figure 1:**
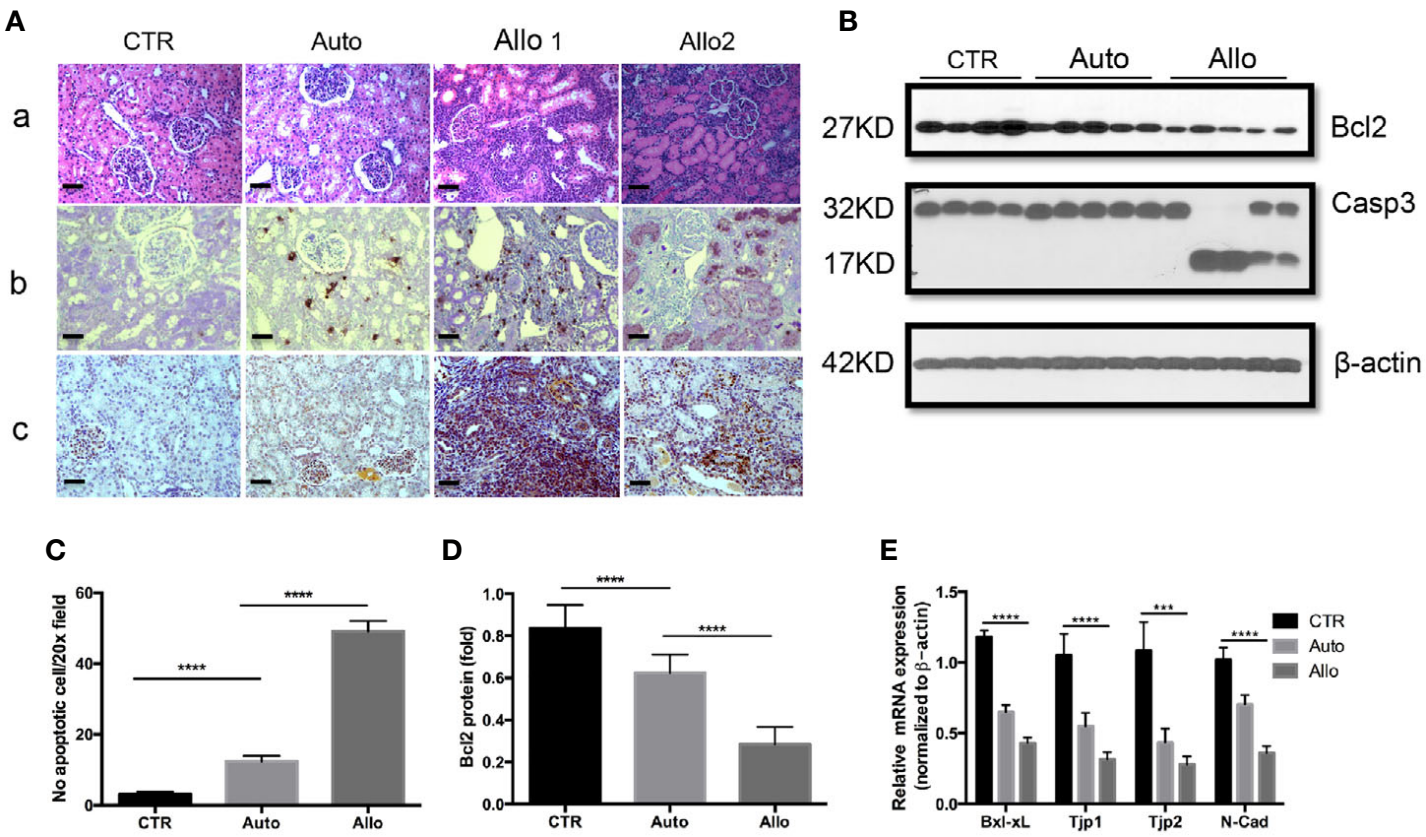
Histology, pathology, and gene expression of kidney tissues. **(A)**: **(a)** Histology of a representative control right kidney (CTR), an autotransplant kidney (auto), and 2 Banff level III rejection allografts (allo). N-18 for the CTR group, n = 5 for the auto group, and n = 18 for the allo group. **(b)** Apoptotic cells of TUNEL positive staining on representative CTR, auto, and allograft. **(c)** Necrotic cells of cyclophilin A (CypA) positive staining on representative CTR, auto, and allograft. **(B)**: Gene expression of anti-apoptotic protein Bcl2 and caspase 3 in representative CTR, auto, and allograft. **(C)** The quantitative average number of apoptotic cells on the 20× microscope view field from random five pictures on each animal tissue. n = 4 for the CTR group, n = 5 for the autotransplant group, n = 7 for the allotransplant group. **(D)** Quantitative analysis of Bcl2 protein expression in representative graft. **(E)** Quantitative PCR of Bcl-xL, tight junction proteins 1 and 2, and N-cadherin mRNA in grafts. ***P < 0.001, ****P < 0.0001, one-way ANOVA, auto *versus* CTR, allo *versus* auto. The scale bar is 50 µm.

Many factors influence the outcome of allografts in KTx. These factors include the results of ischemia–reperfusion which accompanies the surgical procedure and rejection by the recipient’s immune system. Allograft injury is always the first phenomenon in early stage post-transplant before allograft failure and loss. A number of observations have indicated that tubular epithelial cells are the primary targets of IRI and rejection (41–43, 49). Therefore, the alterations of TEC gene profile and cell phenotype as well as TEC death change cell structure and physiological function leading to allograft failure. We first explored cell death patterns in autograft and allograft in pig kidney transplantation. A huge number of cells with positive staining for necrotic biomarker cyclophilin A are seen in allograft rejection within infiltrated recipient circulatory cells and graft’s interstitial structural and tubular cells. No necrotic cells were found in autograft (**Figure 1A**). Interestingly, both H&E staining and TUNEL staining for apoptotic cells indicated specific tubular injury and increasing apoptotic cells in allograft tubules. Apoptotic cells are increased four-fold in autografts and increased 16-fold in allografts (**Figures 1A, C**).

Transplantation procedures were performed on 52 pigs. Among them, 20 pigs underwent allotransplant without any drug treatment. Five of the twenty pigs (two of which were twins) experienced allograft rejection rated IIA on the Banff scale, while six pigs experienced allograft rejection below IIA on the Banff scale. Pigs with allograft rejection rated IIA and lower had normal physiological parameters (40, 50). Nine of the twenty pigs had allograft rejection rated above Banff scale IIA and abnormal physiological function (**Figure 1A**). We picked one pig allograft with Banff scale IIA to group with allografts rated higher on the Banff scale to investigate the expression of apoptotic markers Bcl2 and caspase 3 (Casp 3).

Bcl2 family proteins play an important role in cell apoptosis. Western blot showed that anti-apoptotic Bcl2 is moderately reduced in the autograft and dramatically decreased in allograft. Bcl2 reduction is further associated with the activation of Casp 3 (**Figures 1B, D**). Allografts with Banff II had normal physiological function which was only shown on reduction of Bcl2 but no activation of Casp 3 (**Figure 1B**). Bcl-xL is another anti-apoptotic protein in the Bcl2 family (51). Quantitative real-time PCR indicated that Bcl-xL was greatly decreased in allograft rejection (**Figure 1E**). In addition to cell death, epithelial–mesenchymal transition (EMT) is another phenotype of epithelial cell injury which results in changes in epithelial cell physiological function. Western blot indicated that the biomarker of EMT, N-cadherin (N-cad), was reduced in allograft rejection and was associated with the reduction of tight junction protein 1 (TJP1) and tight junction protein 2 (TJP2) expression (**Figure 1E**).

### Rejection and Biological Function of Allograft Were Associated With Expression of KMO and Its Downstream Genes in Kynurenine Metabolism

**Figure 2:**
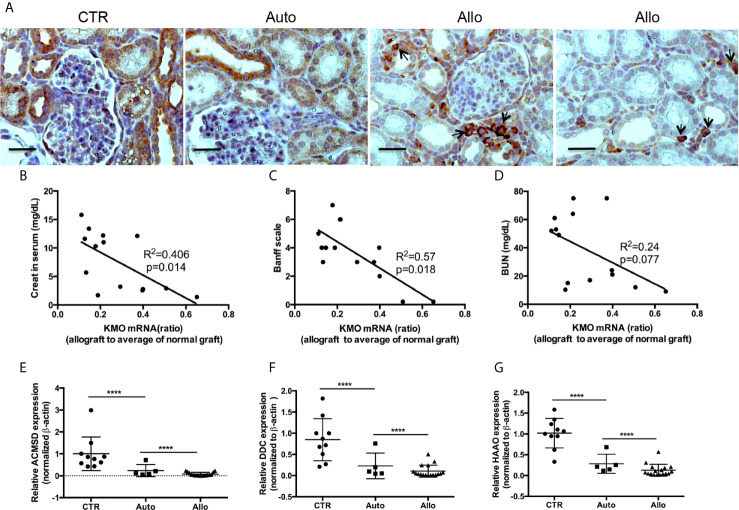
The expression of KMO and other kynurenine metabolic enzymes in grafts. KMO mRNA in grafts were determined by real-time PCR *via* using three pairs of PCR primers which amplify the 5 terminal (161–265 bp), middle (493–600 bp), and 3 terminal (1,239–1,349 bp) specific sequence of KMO gene; the real-time PCR from three pairs of primers showed almost the same result. *β*-actin mRNA for normalization, an average of KMO mRNA was calculated from the results of real-time PCR from 19 normal pig kidneys. The ratio of KMO mRNA in allograft rejection to the average of KMO mRNA in normal kidneys is KMO mRNA fold alterations. **(A)** IHC of KMO on graft tissue section. **(B–D)** Correlation of KMO mRNA ratio in allograft rejection with serum creatinine, allograft Banff scale, and BUN in recipients. **(E–G)** Quantitative analysis of HAAQ, DDC, and ACMSD in grafts. ****P < 0.0001, one-way ANOVA, Auto *versus* CTR, Allo *versus* Auto. The scale bar is 500 µm.

Our recent article in pig KTx demonstrated the dramatic increase of IDO and a decrease of KMO in allograft rejection (40). KMO is a mitochondrial outer membrane protein. Immunohistochemistry (IHC) of KMO on human kidney tissues from antibody providers indicated that KMO was specifically located in tubular epithelial cells. We further confirmed that high expression of KMO was observed in pig kidney tubular cells. However, KMO expression was reduced in autografts and was almost silenced in allograft rejection (**Figure 2A**). Recipient cells that infiltrate the allograft also have a high expression of KMO (arrows in **Figure 2A**). The ratio of KMO mRNA in allograft rejection to average KMO mRNA from 19 normal grafts was positively associated with allograft biological function measured by creatinine (**Figure 2B**) and was negatively associated with the grade of allograft rejection on the Banff scale (**Figure 2C**). However, the ratio of KMO mRNA in allograft rejection to average KMO mRNA from normal grafts was not associated with the BUN level (**Figure 2D**). The expression of more genes in the kynurenine metabolic pathway was analyzed with quantitative PCR. The results indicated that 3-hydroxyanthranilic 3,4 dioxygenase (HAAO), aminocarboxymuconate-semialdehyde decarboxylase (ACMSD), and dopa decarboxylase (DDC) were dramatically reduced in autografts and further decreased in allograft rejection (**Figures 2E–G**).

### Cytokine Storm From IRI and Rejection Induced Gene Expression and Signaling Alterations on TEC

Our previous study showed that many cytokines were over-produced in autografts and allografts (40). IL17 is associated with rejection (52–55). IL17 and IL6 are associated with rejection injury (56–58), while IL18 induces local cytokines and chemokine expression in the allograft (59, 60). Quantitative PCR analysis showed that IL17 expression did not change in autografts but is increased 3.03-fold in the allografts. IL18 increased 1.38-fold in autografts and increased 2.34-fold in the allografts. IL6 increased 2.11-fold in the autografts, but decreased a little in allograft rejection (data not shown). Furthermore, allograft rejection in pig KTx had increased expression of IFN**γ**, TNFα, and IL1β (40). Therefore, allografts are exposed to many altered cytokines or chemokines from ischemia–reperfusion and early rejection procedure.

**Figure 3:**
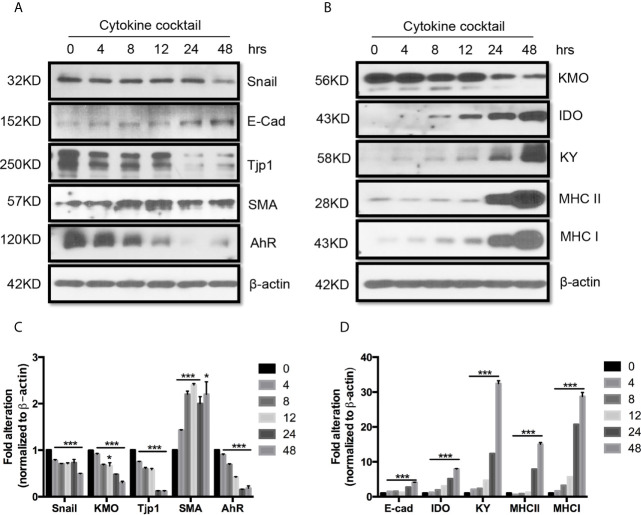
Western blot analysis of function protein expression on primary human renal cortical epithelial cells challenged with cytokines. Primary human renal tubular epithelial cells are challenged with cytokine cocktail (15nM IFN*γ*, 6nM TNFα, and 3nM IL1β). **(A, B)** Expression of Snail, E-Cad, TJP1, SMA. AhR, IDO, KMO, KY, MHCI & II. **(C, D)** Densitometric quantitation of protein in **(A, B)**. All Western blots are representative of at least three independent experiments. *P < 0.05, ***P < 0.0001 *versus* to cells without treatment, analysis is multiple t-tests, pairwise comparison of individual time-point to control indicated same p-value.

**Figure 4:**
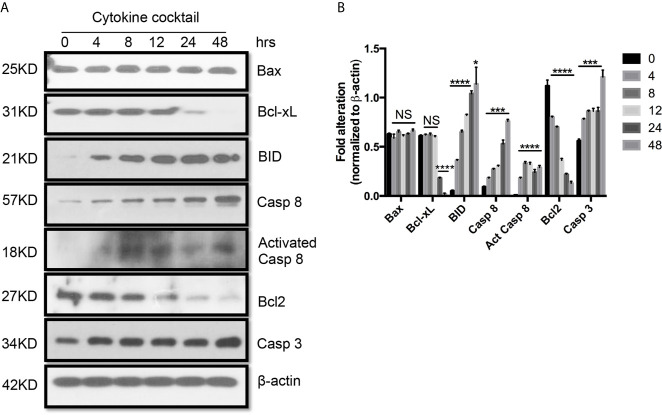
Western blot analysis of apoptotic protein expression on primary human renal cortical epithelial cells challenged with cytokines. **(A)** The expression of Bcl2, Bcl-xL, BID; Bax caspase 3 and 8 (Casp 3 and 8), activated caspase 8 (Act Casp 8) in cells treated with cytokine cocktail. **(B)** Densitometric quantitation of proteins in **(A)**. All Western blots are representative of three independent experiments. NS, no significant, *P < 0.05, ***P < 0.0001, ****P < 0.00001 *versus* cells without treatment; analysis is multiple unpaired t-tests. Pairwise comparison of individual time-point to control indicated the same p-value.

Next, we used TEC to investigate their injury caused by cytokine storm using a cytokine cocktail consisting of IFN**γ**, TNFα and IL1β. Western blot indicated TEC challenged with cytokine cocktail showed increased IDO, KY, and MHC I & II. We also found both E-cadherin (E-cad) and *α*-smooth muscle actin (SMA) increased. KMO, AhR, and TJP1 were greatly decreased. Snail expression was reduced (**Figure 3**). We also found the expression of Bax was unchanged while BID was greatly induced, and Bcl2 and Bcl-xL were dramatically reduced. Both total Casp 3 and caspase 8 (Casp 8) were increased (**Figure 4**). Alterations of Bcl2 family members’ expression and activation of caspases causing epithelial cell apoptosis in allograft rejection confirmed cell apoptosis in allograft rejection *in vivo* (**Figure 1B**).

**Figure 5:**
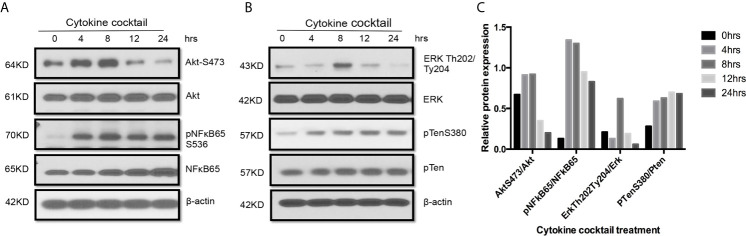
Activation of multiple signaling pathways in TEC challenged by cytokines. Primary human renal TECs were treated with the cytokine cocktail. **(A, B)** Western blot of PI3K–Akt pathway, NFkb pathway, and Erk signaling pathway in cells treated with cytokine cocktail. **(C)** Densitometric quantitation of AktS473, pNFkB65, ErkTh202/Ty204, and pTenS380. All Western blots are representative of two independent experiments.

Western blot also indicated that multiple signaling pathways including AktS473, pNF*κ*B65, ErkTh202/Ty204, and PTenS38 in TEC challenged with cytokine cocktail were activated in different time-points (**Figure 5**). These activated pathways showed the complex biological responses in allograft rejection. Indeed, three signaling pathways can be activated in TEC just from ischemia–reperfusion injury only (43). It is expected that allograft rejection in KTx will activate much more complex biological procedures.

### The 3HK and 3HAA From KMO and Its Downstream Partners in Kynurenine Metabolism Effectively Protected TEC From Cytokines-Induced Injury and Inhibited T Cell Proliferation

**Figure 6:**
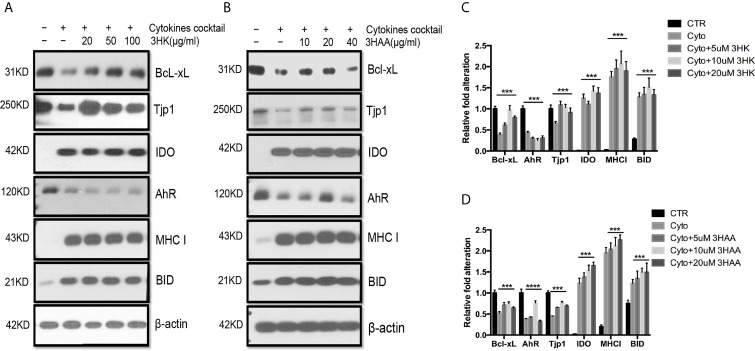
Protective role of 3HK and 3HAA in TEC stimulated with cytokines. Primary human renal TEC pre-incubated with different doses of 3HK or 3HAA overnight; the cells are challenged with the cytokine cocktail (15 nM IFN**γ**, 6 nM TNFα, and 3 nM IL1β) for 24 h; protein expression was assayed with Western blot: **(A, B)** 3HK and 3HAA cannot reverse IDO, MHC I, BID expression induced by cytokines. 3HK and 3HAA effectively restore Bcl-xL and Tjp1 expression. 3HAA shows its different function in up-regulation of AhR expression in TEC in inflammatory conditions. **(C)** Qualification of protein expression in TEC challenged with cytokine cocktail in the presence of 3HK. ***P < 0.0001 *versus* cell treated with Cyto. **(D)** Qualification of protein expression in TEC challenged with cytokine cocktail in the presence of 3HAA. ***P < 0.0001, ****P < 0.00001 *versus* cell treated with Cyto. Pairwise comparison of individual dose to control indicated same p-value. All Western blots are representative of three independent experiments; analysis is multiple one-way ANOVA.

KMO was down-regulated in autografts and was almost completely silenced in allograft rejection (40). KY was also decreased in autografts and further reduced in allograft rejection, negatively affecting the production of 3HK and 3HAA. This contrasts with greatly increased IDO expression in allograft rejection. Upon exposing TEC to the cytokine cocktail in the presence of 3HK and 3HAA, Western blot showed that both 3HK and 3HAA caused up-regulation of Bcl-xL and TJP1 expression (**Figures 6A–C**). 3HAA, but not 3HK, reversed cell tolerance protein AhR expression (**Figure 6A**). These findings suggest that both compounds have a different specific function, and both play crucial roles in protecting TEC from injury.

**Figure 7:**
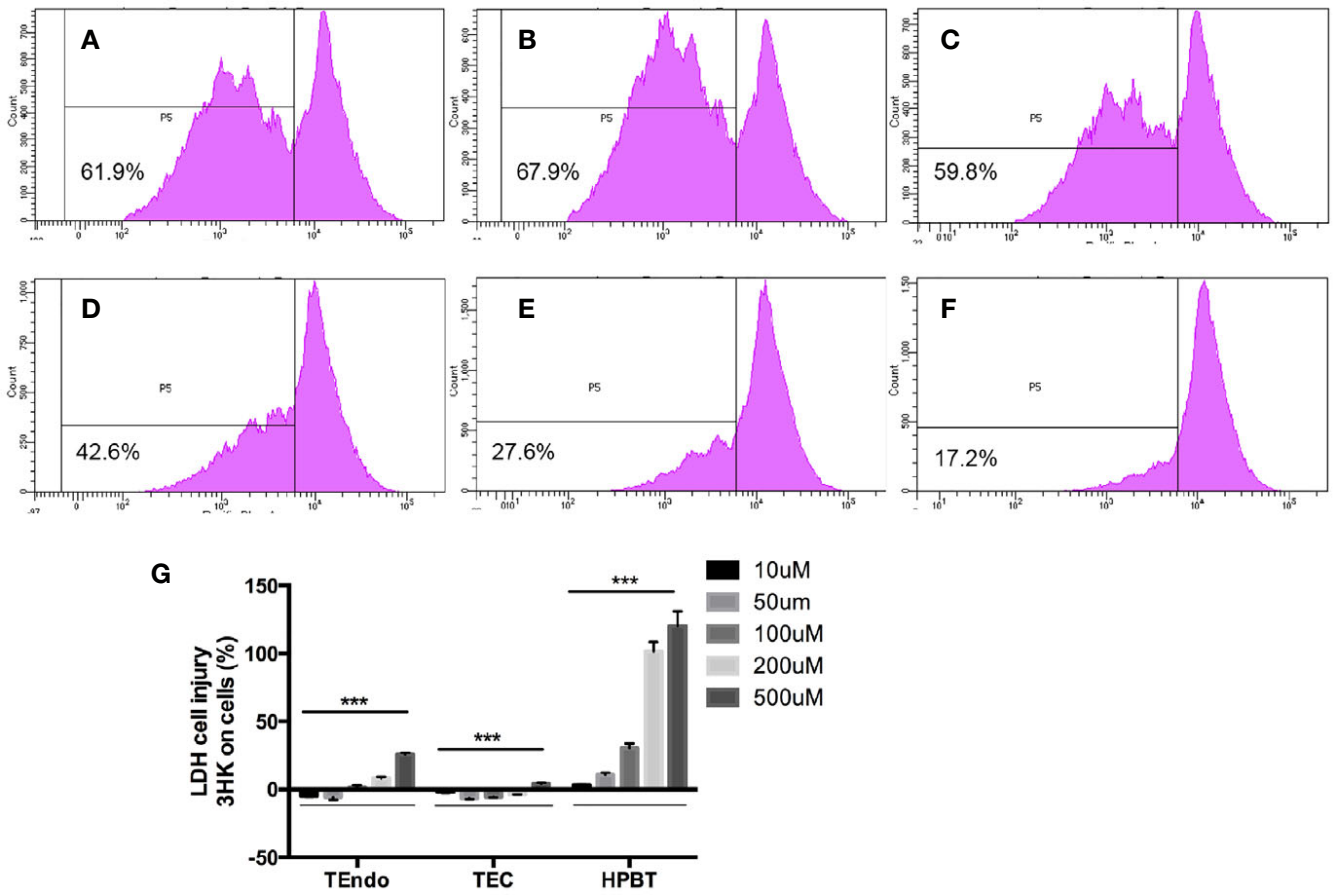
Effects of 3HK and 3HAA on hPBT proliferation and 3HK toxicity on TEndo, TEC, and hPBT. hPBT cell proliferation in **(A)** normal medium, **(B)** cytokine cocktail; **(C)** cytokine cocktail plus 3HK (50 µg/ml); **(D)** cytokine cocktail plus 3HK (100 µg/ml); **(E)** cytokine cocktail plus 3HAA (10 µg/ml) and **(F)** cytokine cocktail plus 3HAA (20 µg/ml); the data of flow cytometry is representative of four independent experiments. **(G)** Cytotoxicity of 3HK on Endo, TEC, and hPBT. The assay is representative of four independent experiments. ***P < 0.001 *versus* cells without treatment; analysis is the unpaired t-tests.

Flow cytometry analysis shown that cytokine cocktail dramatically increased T cell proliferation (**Figures 7A, B**), whereas 3HK and 3HAA effectively inhibited T cell proliferation at 100 and 10uM (**Figures 7C–F**), which confirmed previous observations (24, 25, 61). 3HK is toxic to neurons and pancreatic tissue in high doses (62–64). We performed an LDH assay to address the toxicity of 3HK on TEC, TEndo, and hPBT. We found that 3HK had a protective role on TEC and TEndo at the concentration below 100 µM and is toxic to hPBT even at the concentration of 10 µM (**Figure 7G**).

### DNP-Induced KMO Expression or Increasing 3HK Specifically Reduced Allograft Rejection and Saved Graft Biological Function

**Figure 8:**
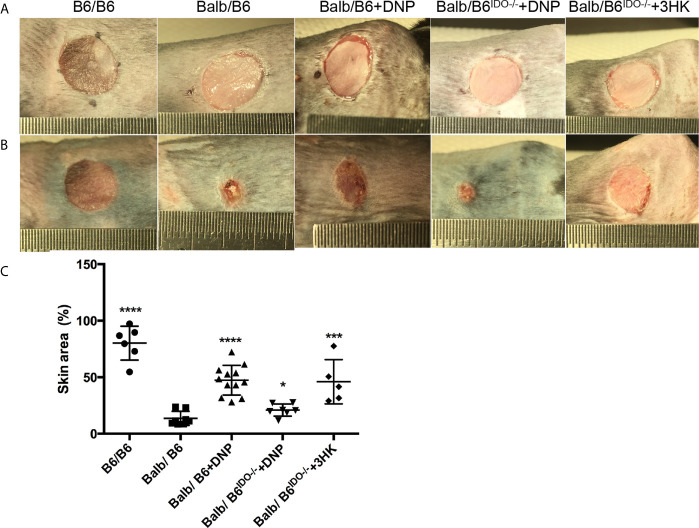
Tolerance effect of kynurenine and its metabolic derivatives in skin graft rejection. **(A)** Eight-week-old B6 or IDO knockout (B6^IDO−/−^, IDO-KO) recipients were transplanted with skin from donor Balb/c (Balbc) mice and treated with DNP or 3HK (i.p. injection) on day 0 (the day before operation). Each panel shows the appearance of donor ear skin tissues immediately following grafting onto the recipient on day 1 (operation day). **(B)** Skin grafts on day 14 from the same mice as **(A)**. **(C)** Graft remaining area in percent on day 14 compared to day 1 from different treatments. *P < 0.05, ***P < 0.001, ****P < 0.0001 *versus* Balb/B6; analysis is multiple one-way ANOVA.

Induced or transgene IDO has been reported to attenuate allograft rejection in rodent transplantation model (27, 30, 31, 33, 34, 65), while tolerance was found to be dependent on kynurenines (36–38). However, IDO was greatly induced in allograft rejection in pig kidney transplantation (40). Thus, we subjected the skin transplant from the ear of mice with balb/c background to the trunk of mice and IDO knockout mice with C57black/6 background. Free CpG-Lacz DNA nanoparticles (DNPs) were reported to induce IDO and mediate Treg-relevant tolerance (48, 66). We performed DNP injection to skin transplanted mice for 13 days post-transplant. DNP effectively attenuated skin allograft rejection in wild-type C57black/6 mice but did not reduce skin allograft rejection on IDO knockout C57Black/6 mice (**Figure 8**). Meanwhile 3HK injection effectively reduced allograft rejection on IDO knockout C57Black/6 mice (**Figure 8**) and wild-type C57black/6 mice (data not shown).

**Figure 9:**
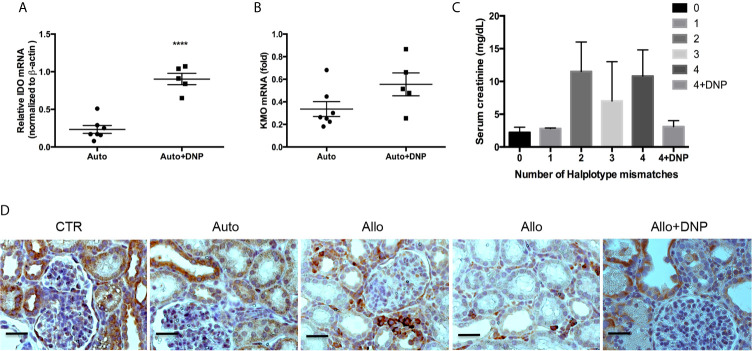
Reduction of allograft rejection by DNP-induced KMO upregulation. Grafts were ex vivo perfused with DNP, transplanted, and harvested at 72hours post-transplantation. **(A)** DNP-induced IDO expression **(B)** DNP-induced KMO expression, **(C)** Effect of DNP treatment on renal function (serum creatinine) from mismatched transplants. **(D)** Representative KMO IHC expression from normal graft right kidney as control (CTR), and autograft (Auto), mismatched allograft (Allo), and mismatched allograft treated with DNP (Allo+DNP). ****p < 0.0001, analysis is multiple unpaired t-tests. The scale bar is 500mm.

We further investigated whether DNP treatment attenuated allograft rejection in a pig kidney transplantation model. *Ex vivo* perfusion of allografts with DNP pretransplantation resulted in a 2.4-fold increase in IDO mRNA (**Figure 9A**), a 2.7-fold increase in IDO protein, and a 2.45-fold increase in IDO activity (data not shown). In association with increased IDO, KMO mRNA was also increased 2.5-fold (**Figure 9B**). The allograft treated with DNP showed less rejection (**Figure 9C**). IHC of KMO also indicated that allografts pretreated with DNP had less cell infiltration, and TEC retained high expression of KMO in graft tubules (**Figure 9D**).

## Discussion

Rejection of renal allograft and acquired complications of long-term immunosuppressant medication aimed at attenuating rejection by the recipient’s immune system are the major causes of subsequent allograft loss. Therefore, investigation of the molecular mechanism of rejection and treatment of rejection has been the foremost subject of interest since the establishment of allogeneic transplantation. Targeted improvements in immunosuppressive therapy could have a substantial impact on patients’ long-term survival. Although immunosuppressants have already improved in terms of specificity and lower toxicity secondary to a better understanding of molecular mechanism and pathology of rejection, the renal complex structure and complicated cell biological function in glomeruli and tubules, especially the cells in different tubular segments, have a different biological function, which restricts the dose and usage of antirejection drugs. In multiple organs or many types of cells in one organ, the drugs may favor some specific cells but injure other cells. Therefore, the overdose or long-term usage of antirejection medication results in a series of complications. Indeed, current clinical use of antirejection drugs in KTx including glucocorticoids, cyclophosphamide, chlorambucil, azathioprine, mycophenolate salts, cyclosporine, and tacrolimus can cause toxicity to allograft and other organs (67–73). Thus immunosuppressants with low toxicity and high efficiency are in high demand. The ideal drugs are metabolites preventing rejection of the transplanted organ while also providing normal physiological function, effectively optimizing the recipient’s immune response with diminished toxicity.

IDO has been reported to prevent allogeneic fetal rejection by depletion of tryptophan or increase the ratio of kynurenine to tryptophan (18). Studies have indicated that induced or transgene IDO can attenuate allograft rejection in rodent transplant models (18, 26–35). Given these findings, we planned to improve the outcome of allograft rejection in pig mismatch kidney transplantation *via* inducing IDO expression. We found that IDO activity increased from 10 to 100-fold without a clear correlation with allograft function. Some allografts with high IDO activity had normal physiological function, while some allografts with high IDO activity had an abnormal function other allografts with low IDO activity lost function. Interestingly, allografts with normal function had low IDO activity and relatively high KMO expression. KMO expression had an inverse association with creatinine concentration in the serum of the recipient holding allograft rejection. This led us to hypothesize that KMO may be the main mediator preventing rejection in IDO-initiating tryptophan to kynurenine metabolites. Indeed, a 2.5 to 5.2-fold kynurenine increase and a 6.6 to 58.3-fold 3HAA increase have been found in cord blood (74). As the intermediate between kynurenine and 3HAA in the kynurenine metabolic pathway, 3HK should play an important role in preventing fetus rejection.

TECs are the primary targets in IRI and acute allograft rejection (43, 75), and they exist in recipient urine as a biomarker of acute rejection in the early stage of KTx (76). However, the molecular mechanism of TEC injury and allograft rejection has not been fully described. We and other scientists found that many increased cytokines in the early allograft rejection stage were associated with TEC injury (40, 77–82). These secreted cytokines should play the important role in inducing TEC injury, which leads to graft failure from epithelial to mesenchymal transition (EMT) (83) or cell death including necrosis and apoptosis (76, 84, 85).

Since a number of cytokines have been found to induce TEC injury, and TEC injury is relevant to rejection (86–88), scientists investigate their relationship in order to provide effective prevention and treatment of allograft rejection. Altered gene expression in the ischemia–reperfusion process may induce reversible injury for allograft organ and tissues or may cause an irreversible chain of injuries which lead to rejection. In this study, three cytokines in low-dose induced TEC injury *via* activation of many signaling pathways; injured cells had an abnormal physiological function, altered cell phenotype, and finally resulting in cell death. These cytokines possibly induced dramatic accumulation of MHC I & II on TEC cell surface to trigger direct and indirect immune response by recipient’s immune response. Additionally, the three cytokines almost silenced KMO expression resulting in the reduction of 3HK, which also inevitably affected the tandem enzymatic reaction of kynureninase together with KMO to produce 3HAA. Although 3HAA can also be produced by the combination of kynureninase and anthranilic acid 3-hydroxylase to biologically avoid KMO involvement in producing 3HAA, a previous study revealed 3HK is a stronger inhibitor of T cell proliferation than 3HAA in physiological condition (21). In addition, 3HK has other important biological functions as the metabolic precursor of kynurenine aminotransferase to produce important biological product xanthurenic acid and downstream metabolites. Our current study also revealed that 3HK is a stronger protector of the epithelial cell barrier (**Figure 6A**). Thus, KMO showed its importance in kynurenine metabolism and kynurenines’ immune regulation.

The novel findings here indicated 3HK and 3HAA prevented TEC from injury *via* increasing Bcl-xL to prevent TEC apoptosis and increasing the expression of tight junction protein 1 to maintain the epithelial cell barrier intact. These findings suggest that reduction of KMO in the ischemia–reperfusion process is responsible for TEC and allograft injury in the early stage of KTx.

Half a century ago, experimental observation on murine skin transplant indicated that graft perfusion with allogeneic RNA attenuated allograft rejection to prolong allograft survival (89). It was further discovered that allograft perfused with any RNA and DNA had a better outcome (90). DNA-nanoparticles (DNPs) increase tolerance by IDO-induced Tregs (48). The free-CpG LacZ DNP administration through blood circulation can improve allograft survival in an IDO dependent manner (48, 66). 3HK and 3HAA effectively reduced rejection through inhibition of T cell proliferation, which suggested that kynurenine derivatives are the real players in preventing rejection. IDO initiates the production of kynurenine, which is further metabolized by downstream enzymes KMO and kynureninase to produce 3HK and 3HAA respectively; both kynurenine derivatives strongly prevent allograft rejection.

*Ex vivo* perfusion of graft pre-transplant with DNP induced IDO and KMO in grafts in a concerted regulation action similar to the molecular regulation of IDO and KMO in the brain (91). KMO was dramatically down-regulated in allograft rejection and TEC in cytokine pool (40); the administration of DNP with *ex vivo* perfusion increased KMO expression associated with reduction of allograft rejection. A previous study revealed increasing KMO may be unfavorable in the brain and pancreas (62–64, 91), but the real risk of pancreatitis from KMO requires further investigation. KMO likely has dual functions that may or may not favor some specific cells (92). Although the reduction of KMO has recently been reported to induce abnormal kidney function (93), the beneficial functions of KMO on other tissues or organs may have been overlooked. The administration of 3HK to the recipient of skin transplant can effectively improve the outcome of allograft on the IDO knockout model; this clearly defines the tolerogenic role of KMO.

When pig grafts were *ex vivo* perfused with DNP, we found that pig grafts are very sensitive to lipid toxicity; the ratio of lipid to DNA is very crucial for attenuation of allograft rejection and improvement of allograft function. Some liposomes and nanomedicine can induce complement activation-related pseudoallergy on pig models (94). The ideal nano-therapeutic should be carefully tested to remove lipid toxicity on drug treatment.

The combination of IFN**γ**–TNFα–IL1β causing TEC injury with increased MHC I and II provides the potential molecular mechanism of rejection on TEC and allograft during transplantation. The expression and regulation as well as location of MHC I and II induced by cytokines should be further investigated in the future, which may decipher the molecular mechanism of allograft rejection from ischemia–reperfusion and early stage of rejection procedure in organ transplantation. In current study, KMO served as a crucial mediator in linking ischemia procedure to rejection as evidenced by its dramatic down-regulation in ischemia–reperfusion resulting in low 3HK and 3HAA production and ultimately leading to loss of TEC self-protection. This allowed disruption of endogenous metabolites in TEC in preventing T cell proliferation with the final consequence of rejection.

The formation, prevention, and outcome of allograft rejection in KTx is a very complex subject, which relies on many factors such as ischemia–reperfusion procedure and operation procedure, instinctive genetic match, and side effect of long-term medication. The early disruption of gene expression will trigger a reaction chain to result in graft rejection and loss. The current study demonstrated that down-regulation of KMO caused by ischemia and early stage of rejection is a key mediator to cause allograft rejection due to reduction of 3HK and 3HAA production. 3HK and 3HAA strongly prevent allogeneic T cell proliferation and effectively protect graft barrier function *via* increasing Bcl-xl and TJP1 expression. In addition, an effective elimination of cytokine storm caused in IRI procedure or blocking cytokine-induced reduction of KMO, and moderate supplementation of 3HK and 3HAA after transplantation can provide graft better outcome.

## Data Availability Statement

The raw data supporting the conclusions of this article will be made available by the authors, without undue reservation.

## Ethics Statement

The animal study was reviewed and approved by Augusta University Institutional Animal Care and Use Committee.

## Author Contributions

YW designed the research. YW, RL, and XF performed the research. XF performed murine skin transplantation. TM performed all pig kidney transplantation. YW analyzed the data and wrote the manuscript. All authors contributed to the article and approved the submitted version.

## Funding

This work was supported in part by grants from the Carlos and Marquerite Mason Charitable Trust and Reserve Research grant fund #S-2444 from Dialysis Clinic. Inc.

## Conflict of Interest

The authors declare that the research was conducted in the absence of any commercial or financial relationships that could be construed as a potential conflict of interest.
